# Daraxonrasib, a pan-RAS inhibitor, selectively inhibits osteosarcomas with activated KRAS by halting AKT signaling and matrix metalloprotease activity

**DOI:** 10.1371/journal.pone.0329946

**Published:** 2025-08-08

**Authors:** Okkeun Jung, Angelene Soto, Andrew L. Wolfe, Shahana S. Mahajan

**Affiliations:** 1 Department of Medical Laboratory Sciences, Hunter College, City University of New York, New York, New York, United States of America; 2 Ph.D. Program in Biology (Molecular, Cellular, and Developmental Biology Sub-Program), The Graduate Center of the City University of New York, New York, New York, United States of America; 3 New York Research and Mentoring for Postbaccalaureates at Hunter College of the City University of New York, New York, New York, United States of America; 4 Department of Biological Sciences, Hunter College of the City University of New York, New York, New York, United States of America; 5 Ph.D. Program in Biochemistry, The Graduate Center of the City University of New York, New York, New York, United States of America; 6 Department of Pharmacology, Weill Cornell Medical College, New York, New York, United States of America; 7 Brain Mind Research Institute, Weill Cornell Medical College, New York, New York, United States of America; Zhejiang Normal University, CHINA

## Abstract

KRAS mutations, which induce proliferative signaling driving many human cancers, are detectable in a small subset of osteosarcoma patients. The recently developed pan-KRAS inhibitor daraxonrasib, also known as RMC-6236, is capable of targeting a wide array of KRAS mutations and shows promise against pancreatic and lung cancers. However, the efficacy and mechanisms of action of daraxonrasib in osteosarcoma (OS) remain unclear. We evaluated the effects of daraxonrasib on the viability, proliferation, and metastatic potential of wild-type and KRAS mutant OS cells. We assayed the effects of treatment on downstream targets using qPCR, immunoblotting, and activity assays to explore the underlying mechanism by which daraxonrasib selectively suppresses the metastatic potential of KRAS mutant osteosarcoma. Finally, we investigated how the increased prevalence of GTP-bound KRAS enhanced the sensitivity of KRAS wild-type osteosarcoma cells to daraxonrasib using siRNA targeting RASA1. Daraxonrasib selectively attenuated the proliferation and migratory ability of KRAS mutant HOS-143B cells without affecting KRAS wild-type controls. Additionally, daraxonrasib suppressed the expression of the matrix metalloproteases MMP9 and MMP1, which promote cell motility and metastasis. Daraxonrasib selectively inhibited the AKT/ETS1 pathway in HOS-143B cells, whereas no such effect was observed in HOS cells. HOS cells were sensitized to daraxonrasib by knocking down the GTPase-activating protein RASA1. In osteosarcoma, KRAS inhibition decreased MMP1, MMP9, and AKT/ETS1 signaling. Daraxonrasib is a promising agent for treating osteosarcoma with KRAS mutations.

## Introduction

Osteosarcoma (OS) is a malignant bone tumor that predominantly occurs during adolescence. Despite the development of anti-cancer drugs targeting osteosarcoma, the survival rate of patients with metastatic osteosarcoma has not improved [1].

KRAS is a growth-regulatory GTPase that is frequently mutated in many cancers. Although oncogenic KRAS mutations are rare in human osteosarcoma, they have been previously reported [2,3]. Oncogenic KRAS G12D and Q61L mutations have been identified in Ewing’s sarcoma samples [4–6]. KRAS mutations are most commonly found in non-small cell lung cancer (NSCLC), colorectal cancer, and pancreatic cancer [7,8]. Currently, KRAS G12C is the only mutation that has been successfully targeted using a clinically approved KRAS inhibitors. KRAS G12C inhibitors thus far have only been approved for use as a monotherapy in non-small cell lung cancer and as a combination therapy in colorectal cancer, partially because of the rarity of the G12C allele and partially because of a lack of drug sensitivity in certain tumor types. The extent to which KRAS-mutant osteosarcoma cells are sensitive to KRAS inhibitors remains to be determined.

Several experimental compounds currently in development show promise for targeting other KRAS mutant alleles [9–11]. Daraxonrasib, also called RMC-6236, is a recently developed pan-RAS inhibitor that targets KRAS in a GTP-bound state irrespective of the driver mutation. Daraxonrasib is a non-covalent inhibitor originally developed from sanglifehrin A, which has a cyclophilin A binding region and a RAS binding region, allowing it to act as a scaffold for a tri-complex that occludes RAS effector binding sites (S1A Fig) [12]. KRAS inhibition leads to decreased binding to its effector RAF1, typically leading to decreased p-ERK and cell proliferation [13]. Daraxonrasib halts growth of KRAS-addicted lung cancer and colon cancer cells; however, its efficacy and mechanisms of action in bone cancers have not been evaluated [9,10].

Metastasis affects nearly 90% of cancer patients and is a significant factor in the high mortality rate [14]. In order for cancer to metastasize to other tissues, extracellular matrix (ECM) must initially be degraded, a process facilitated by the secretion of matrix metalloproteases (MMPs) [15]. The ECM consists of molecules that surround tissues and organs, providing structural support and serving as a physical barrier [16].

In this study, we employed two osteosarcoma cell lines, HOS and HOS-143B, to understand the mechanism of how daraxonrasib affects osteosarcoma with KRAS WT versus the oncogenic mutation KRAS G12S [17]. The HOS-143B cell line was established by infecting the HOS cell line with Kirsten mouse sarcoma virus (Ki-ras^+^) [18]. Here, we found that treatment with the pan-RAS inhibitor daraxonrasib led to dose-dependent inhibition of growth and migration in the KRAS G12S osteosarcoma cell line HOS-143B, while having virtually no effect on KRAS wild-type osteosarcoma line HOS. Daraxonrasib selectively inhibited MMP1, MMP9, and AKT/ETS1 signaling in HOS-143B cells. These findings support the therapeutic potential of daraxonrasib as an anti-cancer agent for KRAS-mutant osteosarcoma.

## Results

### Daraxonrasib suppresses the proliferation of KRAS mutant osteosarcoma

**Fig 1:**
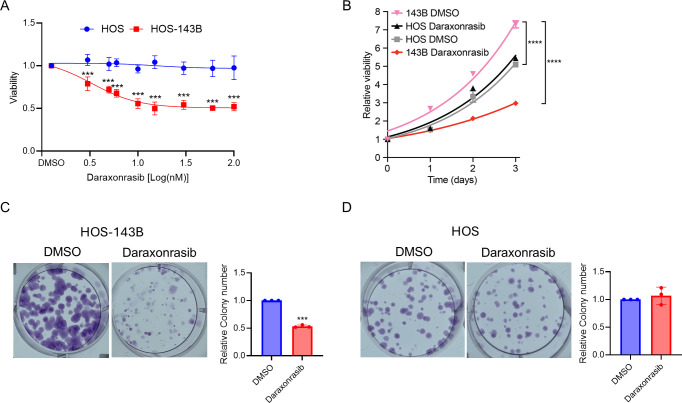
Pan-KRAS inhibition selectively halts proliferation of KRAS G12S HOS-143B cells. (A) Cytotoxicity of daraxonrasib in HOS-143B and HOS cells. (B) Proliferation assay across three days of treatment with DMSO or 5 nM daraxonrasib in HOS and 143B cell lines. (C-D) Colony forming assay and quantification of colony numbers of (C) HOS-143B and (D) HOS. All experiments were conducted independently in triplicate. Data are presented as the means ± SD. Student’s t-test was conducted to assess the significance of two-group comparisons. For comparisons involving more than three groups, one-way ANOVA was performed, followed by Dunnett’s test for post-hoc analysis to determine statistical significance. *p < .05, **p < .01, ***p < .001, ****p < .0001.

We initially measured basal levels of KRAS in both HOS and HOS-143B (143B) cells, confirming that KRAS is overexpressed in HOS-143B cells (S1B Fig). To address the effect of daraxonrasib on KRAS WT and mutant osteosarcoma, we examined the cytotoxicity of daraxonrasib in HOS and 143B cell lines. The results of both dose-response experiments indicated that daraxonrasib inhibited HOS-143B cells at all tested doses but did not significantly affect the viability of HOS cells (Fig 1A). This observation suggested that daraxonrasib specifically suppressed the viability of cells with KRAS mutations. To determine whether daraxonrasib affected proliferation over time, we treated HOS-143B and HOS cells with 5 nM daraxonrasib or DMSO for 3 days. As shown in Fig 1B, daraxonrasib significantly inhibited the proliferation of HOS-143B cells. In contrast, HOS cell growth was not suppressed by daraxonrasib. The basal proliferation rate of HOS-143B cells was significantly higher than that of HOS cells (Fig 1B). Next, we examined the long-term anti-proliferative effects of daraxonrasib on both cell lines by performing a colony formation assay over eight days. Treatment with daraxonrasib significantly reduced colony number of HOS-143B cells, while not affecting HOS cells (Figs 1C and D). These results imply that daraxonrasib selectively inhibits the growth of mutant KRAS osteosarcoma.

### Daraxonrasib selectively inhibits metastatic potential of HOS-143B cells

**Fig 2:**
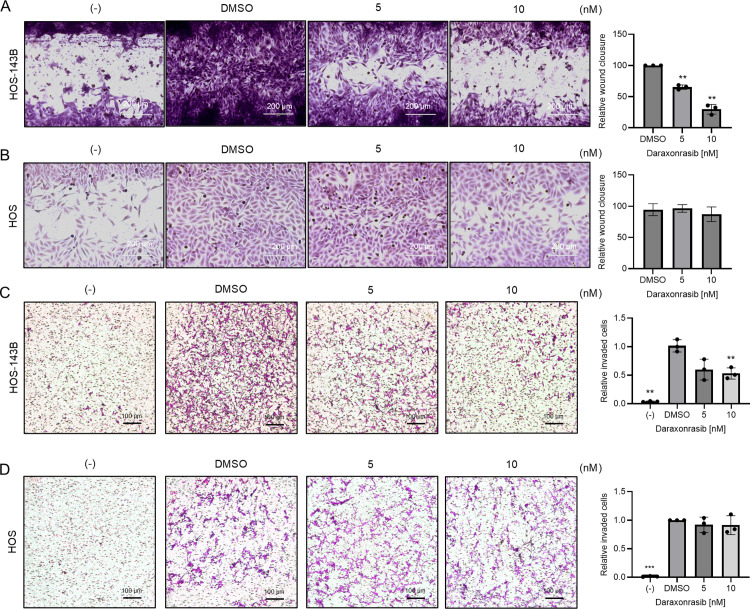
Daraxonrasib selectively impedes the metastatic ability of HOS-143B cells. Wound healing assays were performed to examine migratory ability of (A) HOS-143B and (B) HOS cells. Both cell lines were seeded on 96-well plates, plates were scratched using a 200 µl pipette tip, and cells were incubated overnight with either DMSO or daraxonrasib (5 nM and 10 nM). Media containing 0.1% serum was used as a negative control (-). The negative control shows the initial wound distance. (C-D) A Boyden chamber invasion assay was conducted to access the invasive abilities of (C) HOS-143B and (D) HOS cells. Scale bars represent 200 µm and 100 µm for the wound healing assay and Boyden chamber invasion assay, respectively. All experiments were conducted independently in triplicate, and the data are presented as the means ± SD. One-way ANOVA was performed, followed by Dunnett’s test for post-hoc analysis to determine statistical significance. *p < .05, **p < .01, ***p < .001.

To investigate whether daraxonrasib has an anti-cancer effect, we performed wound healing and invasion assays (Fig 2). Notably, daraxonrasib selectively inhibited the migratory and invasive abilities of HOS-143B cells (Figs 2A and 2C). The invasive abilities of HOS cells remained unchanged following daraxonrasib treatment (Figs 2B and 2D). These results suggested that daraxonrasib suppresses pathways relevant to cancer metastasis in a KRAS mutation-selective manner.

### Daraxonrasib inhibits MMP-9 activity in HOS-143B

**Fig 3:**
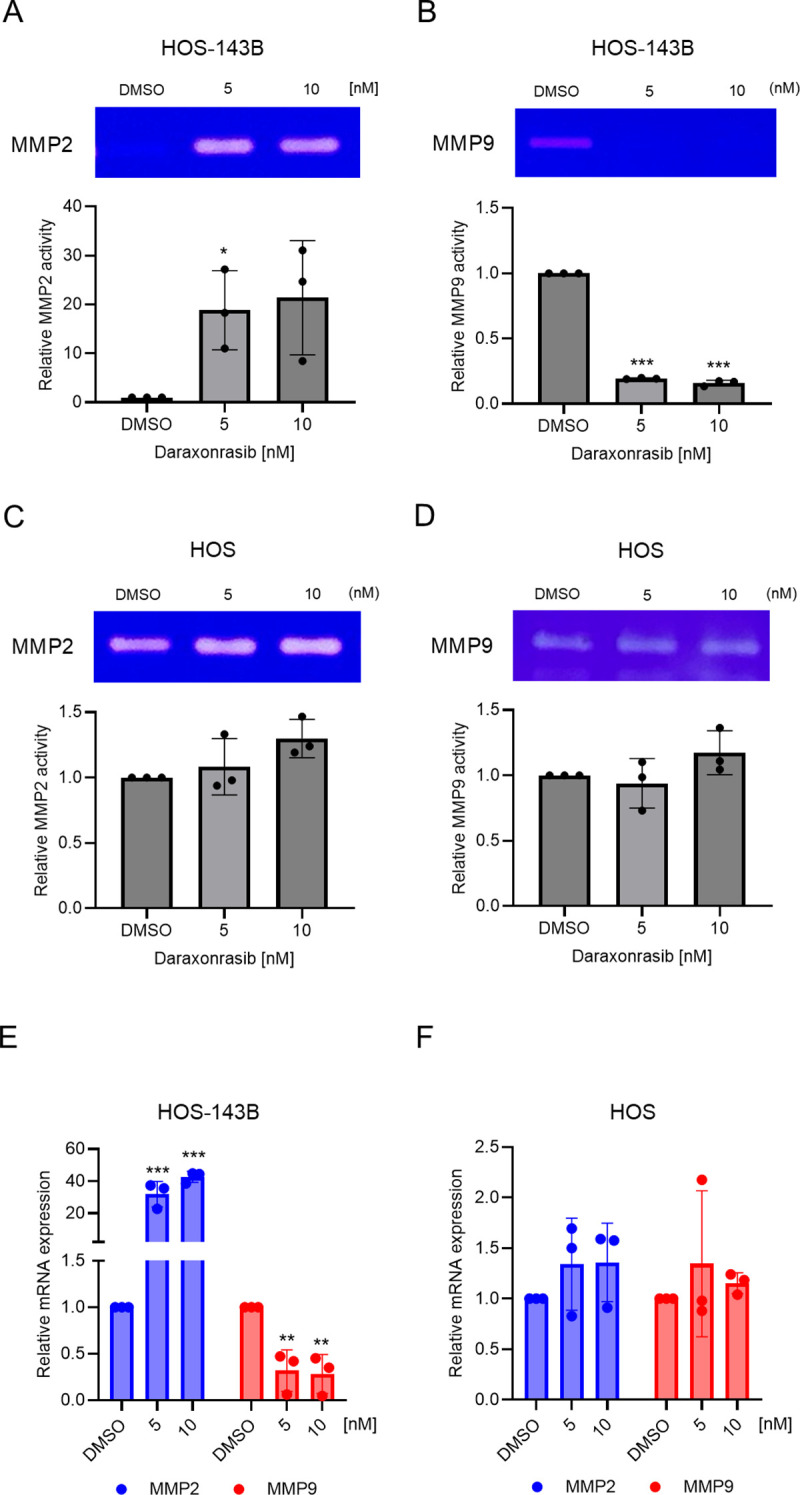
Daraxonrasib inhibits MMP9 expression while upregulating MMP2 expression in HOS-143B cells. Gelatin zymography demonstrated that enzymatic activities of (A) MMP2 and (B) MMP9 in HOS-143B were affected by daraxonrasib. The activities of MMP2 (C) and MMP9 (D) in HOS cells remained unchanged following daraxonrasib treatment. (E) qPCR for MMP2 or MMP9 demonstrated that daraxonrasib upregulated MMP2 mRNA expression while suppressing MMP9 mRNA expression in HOS-143B. (F) Daraxonrasib had no effect on MMP2 and MMP9 mRNA expression in HOS. All experiments were conducted independently in triplicate. The data are presented as the means ± SD. One-way ANOVA was performed, followed by Dunnett’s test for post-hoc analysis to determine statistical significance. *p < .05, **p < .01, ***p < .001.

Upregulation of MMP9 by activation of the PI3K/AKT pathway has been demonstrated to promote osteosarcoma migration [19]. In addition, MMP2 and MMP9 have been proposed as markers for metastasis and angiogenesis [20]. To determine whether daraxonrasib inhibits MMP2 and MMP9 activities, we conducted gelatin zymography in the presence or absence of daraxonrasib (5 and 10 nM). MMP2 activity was upregulated by daraxonrasib in HOS-143B cells (Fig 3A), whereas MMP9 activity was significantly inhibited by daraxonrasib (Fig 3B). Neither MMP2 nor MMP9 activities were altered by daraxonrasib in HOS cells (Figs 3C and D).

Next, we investigated whether daraxonrasib affected the mRNA levels of MMP2 and MMP9. Similar to its effect on MMP2 and MMP9 activities, daraxonrasib significantly increased MMP2 mRNA expression and significantly decreased MMP9 mRNA expression in HOS-143B cells (Fig 3E), while having no effect on MMP2 or MMP9 mRNA expression in HOS cells (Fig 3F).

### Daraxonrasib dramatically inhibits MMP1 activity in HOS-143B

**Fig 4:**
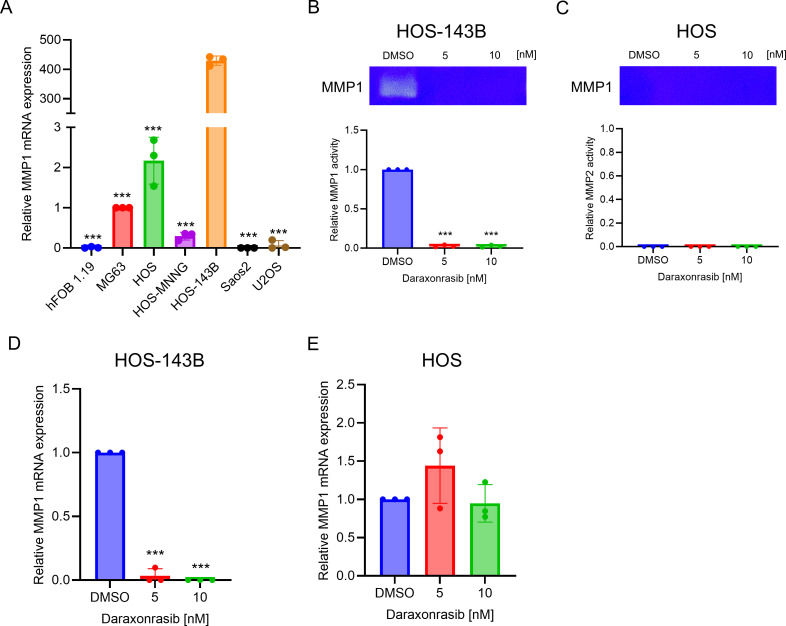
Daraxonrasib effectively suppresses MMP1 expression in HOS-143B cells. (A) comparison of MMP1 mRNA expression in osteosarcoma cell lines. (B-E) Gelatin zymography demonstrated that daraxonrasib inhibited MMP1 activity in (B) HOS-143B, while the MMP1 activity remained unchanged in (C) HOS cells. (D) qPCR demonstrated that daraxonrasib downregulated MMP1 mRNA expression in HOS-143B. (E) Daraxonrasib does not alter the MMP1 mRNA expression in HOS cells. All experiments were conducted independently in triplicate. The data are presented as the means ± SD. One-way ANOVA was performed, followed by Dunnett’s test for post-hoc analysis to determine statistical significance. An asterisk (*) in (A) indicates a comparison of 143B expression to each osteosarcoma cell line. *p < .05, **p < .01, ***p < .001.

MMP1 activity has been reported to stimulate angiogenesis and endothelial cell proliferation through upregulation of VEGFR2 [21]. HOS-143B has also been shown to exhibit high expression of MMP1 and MMP9 [22]. Therefore, we evaluated MMP1 mRNA expression in seven osteosarcoma cell lines, including HOS and HOS-143B. MMP1 showed the highest expression in HOS-143B cells among all osteosarcoma cell lines (Fig 4A). To determine whether daraxonrasib affected MMP1 expression, we examined MMP1 activity using collagen zymography. The results indicated that MMP1 was significantly downregulated by daraxonrasib in HOS-143B cells, but not in HOS cells (Figs 4B and C). In addition, we evaluated mRNA of MMP1 in HOS and HOS-143B cells and confirmed that MMP1 mRNA was selectively suppressed only in HOS-143B cells (Figs 4D and E).

### Daraxonrasib attenuates AKT/ETS1 signaling in HOS-143B

**Fig 5:**
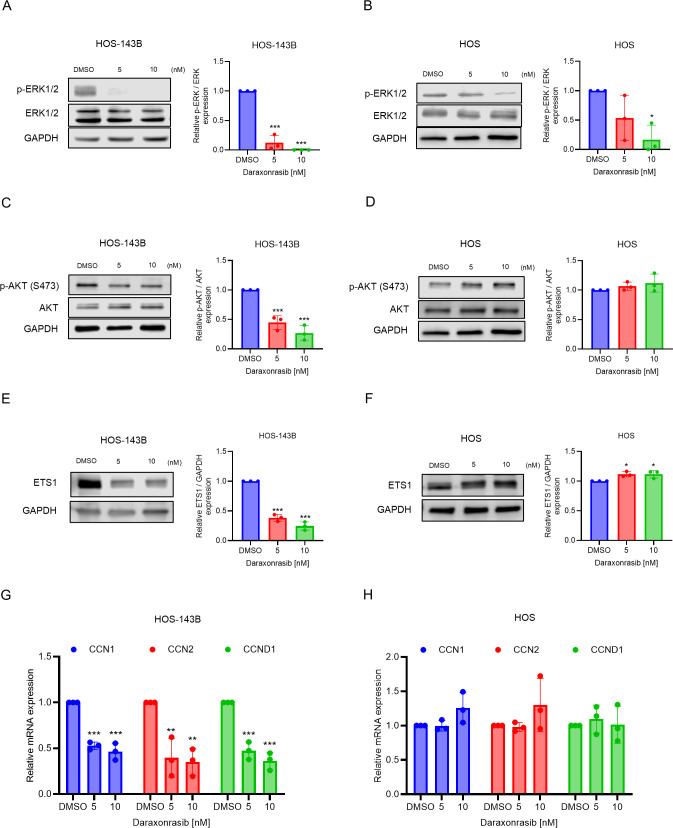
Effects of daraxonrasib on p-ERK, p-AKT and ETS1 in HOS-143B and HOS cells. (A-B) Cells were treated with daraxonrasib (5 nM and 10 nM) for 24 hours. Daraxonrasib treatment downregulated ERK1/2 phosphorylation in both (A) HOS-143B and (B) HOS cells. (C) Daraxonrasib treatment reduced AKT phosphorylation in 143B cells. (D) AKT phosphorylation remained unaffected in HOS cells. (E) ETS1 is downregulated by daraxonrasib treatment in HOS-143B. (F) Daraxonrasib does not attenuate ETS1 levels in HOS cells. (G-H) qPCR for 143B (G) or HOS (H) cells treated with the indicated doses of daraxonrasib for 24 hours. All experiments were conducted independently in triplicate. The data are presented as the means ± SD. One-way ANOVA was performed, followed by Dunnett’s test for post-hoc analysis to determine statistical significance. *p < .05, **p < .01, ***p < .001.

Daraxonrasib recruits CYPA to KRAS to block KRAS interaction with downstream effectors, such as RAF and PI3K [23]. Thus, we hypothesized that daraxonrasib selectively suppresses the invasive ability of HOS-143B cells by suppressing the RAF/MEK/ERK and PI3K/AKT signaling axes [9,24]. Daraxonrasib reduced the active phosphorylated form of ERK1/2 in HOS-143B cells (Fig 5A). Strikingly, at higher doses daraxonrasib inhibited the active form of ERK1/2 in HOS cells as well (Fig 5B).

ETS1 is a metastasis-regulating transcription factor regulated by AKT in NSCLC [25]. Daraxonrasib treatment suppressed phosphorylation of AKT and the expression of ETS1 in HOS-143B cells, while no change was observed in HOS cells (Fig 5 C-F).

### Daraxonrasib selectively inhibits the transcriptional activity of ETS1 in HOS-143B

Next, we hypothesized that daraxonrasib-mediated downregulation of ETS1 would attenuate the transcriptional activity of ETS1-regulated target genes. To evaluate transcriptional activity of ETS1, we performed qPCR and examined expression of three previously reported ETS1 targets involved in promoting cell proliferation: CCN1, CCN2, and CCND1 [25–27]. The results demonstrated that these genes were downregulated by treatment with daraxonrasib in HOS-143B (Fig 5G). Daraxonrasib did not change mRNA expression of these genes in HOS cells (Fig 5H). These results suggest that daraxonrasib inhibits AKT/ETS1 signaling in KRAS mutant cells, resulting in suppression of CCN1, CCN2, and CCND1 expression.

### The inhibitory effect of daraxonrasib depends on the duration of active KRAS persistence

**Fig 6:**
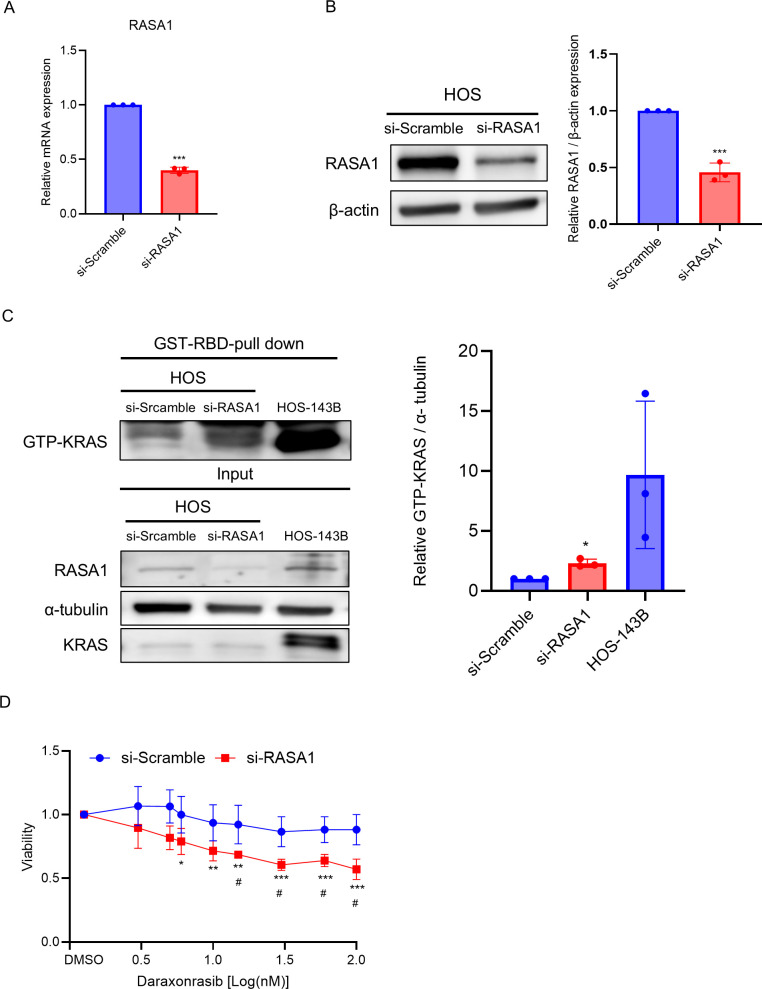
A higher proportion of active KRAS exhibit greater sensitivity to daraxonrasib. (A) qPCR analysis of *RASA1* mRNA and (B) protein expression of RASA1 following treatment with a siRNA targeting RASA1. (C) The effect of *RASA1* silencing on GTP-bound KRAS levels was assessed in HOS cell line. GTP-KRAS expression was evaluated using HOS-143B cells as a positive indicator. (D) Dose response relative viability for cells treated with siRASA1 or a Scrambled siRNA negative control. All experiments were conducted independently in triplicate, and the data are presented as the means ± SDs. One-way ANOVA was performed, followed by Dunnett’s test for post-hoc analysis to determine statistical significance. Student’s t-test was used to determine the statistical significance between the si-scramble and si-RASA groups at the same dosage. Asterisk (*) indicates comparison to DMSO, Hash (^#^) indicates comparison to si-scramble at same dosage. *p < .05, **p < .01, ***p < .001. ^#^*p* < .05, ^##^*p* < .01, ^###^*p* < .001.

KRAS mutations enable cancer cells to proliferate by maintaining a higher proportion of KRAS in the active GTP-bound state [28]. Thus, we hypothesized that an increase in active KRAS may sensitize osteosarcoma cells with WT KRAS to inhibition by daraxonrasib. To test this hypothesis, we used siRNA to silence RASA1 expression in HOS cells. RASA1 encodes the protein p120-RasGAP, a GTPase-activating protein (GAP) that promotes the conversion of active KRAS-GTP into its inactive form KRAS-GDP via hydrolysis [29]. RASA1 expression was efficiently suppressed by si-RASA1 at both the mRNA (Fig. 6A) and protein levels (Fig. 6B). To investigate whether RASA1 knockdown leads to increased GTP-bound KRAS levels in HOS cells, we performed GTP-KRAS pull-down assay. The results showed that the suppression of RASA1 significantly increased GTP-KRAS levels compared to the si-Scramble in HOS cells (Fig 6C). Under these conditions, we evaluated the cytotoxicity of daraxonrasib using si-Scramble and si-RASA1. Intriguingly, daraxonrasib significantly inhibited the viability of HOS cells transfected with an siRNA against RASA1 in a dose-dependent manner, whereas no effect was observed in HOS cells transfected with si-Scramble (Fig 6D).

## Discussion

Single-agent basket clinical trials of the KRAS G12C inhibitors adagrasib and sotorasib led to approval in NSCLC, but not in other diseases [11,30–33]. Daraxonrasib is currently in two Phase 3 clinical trials, one for NSCLC and another for pancreatic ductal adenocarcinoma (PDAC). Bone cancers have not been traditionally well represented in KRAS inhibitor clinical trials because of the low frequency of KRAS mutations and the infrequency of G12C. The recent expansion of the range of druggable KRAS alleles has opened new clinical opportunities to treat rare KRAS-mutant bone cancers.

Here, we discovered that daraxonrasib selectively inhibits the invasive and proliferative abilities of osteosarcoma cells with KRAS mutations. In wound healing assays, we observed some inhibition of the metastatic ability of HOS cells by daraxonrasib at a concentration of 10 nM, although the effect was not significant (Fig 2B). It is conceivable that osteosarcoma with KRAS WT can still be affected by daraxonrasib, and that increasing concentrations of daraxonrasib may inhibit the invasive ability of osteosarcomas with wild-type KRAS. In addition, we confirmed that MMP1 and MMP9 were regulated by daraxonrasib only in 143B-HOS cells. It has been reported that MMP1 and MMP9 are regulated by ETS1 activation [34–36]. Therefore, the inhibition of MMP1 and MMP9 may result from the suppression of AKT/ETS1 signaling.

Several natural compounds have demonstrated anti-cancer effects in NSCLC and prostate cancer through concurrent inhibition of MMP2 and MMP9 expressions [37,38]. Notably, daraxonrasib treatment resulted in suppressed MMP9 expression while concurrently increasing MMP2 expression. This dichotomous effect of daraxonrasib on MMP2 and MMP9 suggests the involvement of a distinct regulatory mechanism influenced by KRAS inhibition. Mutant KRAS halts calcium influx by remodeling STIM expression in colorectal cancer cells [39]. Thus, KRAS inhibition by daraxonrasib may lead to increased intracellular calcium levels, potentially activating calcium-dependent signaling pathways such as CREB [40–42]. Activation of cyclic AMP response element binding protein (CREB) has been reported to induce MMP2 expression in cholangiocarcinoma [40]. In contrast, MMP9 expression, particularly in neutrophils, has been shown to be IL-8–mediated and independent of intracellular calcium levels. Therefore, the selective upregulation of MMP2 observed following daraxonrasib treatment may be attributed to calcium-mediated mechanisms triggered by KRAS inhibition. Further studies are needed to elucidate the mechanism by which daraxonrasib upregulates MMP2 while concurrently suppressing MMP9.

Since daraxonrasib selectively inhibited the cytotoxicity and metastatic ability of HOS-143B, it was expected to selectively inhibit the phosphorylation of ERK1/2 in osteosarcoma cells with mutant KRAS. Remarkably, we also observed suppression of p-ERK1/2 in HOS cells at higher doses (Fig 5B). Taken together, these results suggest that daraxonrasib may inhibit proliferation and metastasis via a pathway other than ERK1/2 suppression. Evaluation of the basal level of KRAS in both HOS and HOS-143B cells indicated that KRAS was significantly higher in HOS-143B cells (S1B Fig). Therefore, the metabolic system and proliferation of HOS-143B cells might be optimized by KRAS overexpression compared to HOS cells, leading to increased vulnerability and proliferation in response to daraxonrasib. We further demonstrated that daraxonrasib inhibition of osteosarcoma depended on the ratio of the active form of KRAS, as demonstrated by the increased daraxonrasib sensitivity of HOS cells that was induced by knockdown of RASA1. (Fig 6D).

**Fig 7:**
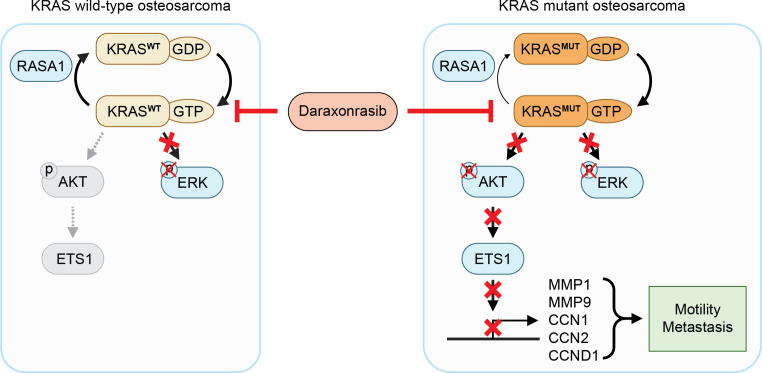
The proposed mechanism by which daraxonrasib exerts its effect involves inhibition of the active form of KRAS, which in turn suppresses AKT/ETS signaling, resulting in the downregulation of MMP1, MMP9, CCN1, CCN2, and CCND1 expression. Normal status of (A) HOS cells and (B) HOS-143B cells. Effects of daraxonrasib treatment on (C) HOS cells and (D) HOS-143B cells.

Based on the present findings, we propose a mechanism by which daraxonrasib suppresses the proliferation and metastatic ability of KRAS mutant osteosarcoma cells by inhibiting overactive AKT/ETS1 signaling (Fig 7). The targeted anti-cancer effect of daraxonrasib highlights its potential as a therapeutic agent for osteosarcoma patients with KRAS mutations. WT KRAS tumors with GAP deficiencies that induce an elevated proportion of active GTP-bound KRAS may be sensitive to daraxonrasib, a possibility that could further expand the use of daraxonrasib for new patient populations with osteosarcoma and beyond.

## Materials and methods

### Compounds

Daraxonrasib was obtained from MedChem Express. The structure of daraxonrasib was drawn using ChemDraw.

### Cell culture

hFOB 1.19 (CRL-3602), MG63 (CRL-1427), HOS (CRL-1543), HOS-MNNG (CRL-1547), HOS-143B (CRL-8303), Saos2 (HTB-85), and U2OS (HTB-96) were purchased from ATCC. hFOB 1.19 cells were incubated in DMEM without phenol red containing 10% FBS, and 1% penicillin and streptomycin. U2OS and Saos2 cells were incubated in McCoy’s 5A without phenol red containing 10% FBS and 1% penicillin and streptomycin. MNNG, MG63 and HOS cells were incubated in EMEM containing 10% FBS, and 1% penicillin and streptomycin. HOS-143B cells were incubated in DMEM containing 10% FBS, BrdU, and 1% penicillin and streptomycin. Cells were maintained in a humidified atmosphere of 37°C, and 5% CO_2_.

### Proliferation assay

Cells were seeded in 96-well plates at a density of 1.0 × 10^4^ cells per well for 24-hour treatment. The cells were treated with DMSO or various doses of daraxonrasib (3, 5, 6, 10, 15, 30, 60, or 100 nM). After treatment, the cells were incubated with MTT solution (5 mg/ml) for 1 h. The media was then replaced with DMSO. Colorimetric changes were measured at 570 nm using a microplate reader.

To investigate the long-term effect of daraxonrasib on HOS and HOS-143B cells, 3 × 10^3^ of HOS or HOS-143B cells were seeded into four separate 96-well plates. On the following day, cells were treated with either DMSO or 5 nM daraxonrasib. One plate was subjected to MTT assay immediately after treatment (Day 0). Subsequently, MTT assay was performed on one plate at the same time each day. All MTT values were normalized to those of Day 0.

### Colony formation assay

Two hundred HOS and HOS-143B cells were seeded in 6-well plates. The following day, the cells were treated with DMSO or 5 nM daraxonrasib for eight days. On the fourth day, the media was replaced with fresh media with DMSO or daraxonrasib. After eight days, cells were fixed with methanol at – 20°C for 1 h. Fixed cells were stained with 0.1% crystal violet solution containing 20% methanol. Stained cells were washed twice with PBS. The number of colonies was quantified using ImageJ software.

### Wound healing migration assay

HOS and HOS-143B cells were seeded in a 96-well plate at densities of 1.2 × 10^4^ cells per well and 1.5 × 10^4^ cells per well, respectively. The following day, the center of each well was scraped using a 200 µl micropipette tip. The wells were washed with PBS and treated with culture media containing either DMSO, or daraxonrasib at 5 nM or 10 nM. Media containing 0.1% FBS was used as a negative control. The following day, cells were fixed with methanol and incubated at −20°C for 1 h. After fixation, the cells were stained with 0.1% crystal violet and the wound area was imaged under a microscope. For each image, the gap was measured at three different points and averaged. These average values were used to evaluate the effect of daraxonrasib on the migratory abilities of HOS and HOS-143B cells.

### Boyden chamber invasion assay

The polycarbonate membrane with 8 µm pore size was coated with gelatin coating solution (0.1% acetic acid and 0.02% gelatin) for 1 h. Media containing 0.1% FBS was used as a negative control. The media containing 1% FBS with either DMSO or daraxonrasib (5 or 10 nM) was added to the lower chamber. Then, the gelatin-coated membrane and upper chamber were placed on top. Cells were collected and resuspended in 0.1% FBS media. The cell suspension was then added to the upper chamber and incubated in a cell culture incubator. After incubation, the membrane was fixed with methanol at −20°C for 2 h. Following fixation, the membrane was stained with 0.1% crystal violet solution. The membrane facing the upper chamber was gently wiped with Kimwipes soaked in 30% glycerol to remove non-invading cells. The invading cells were imaged using a microscope (Zeiss, Scipe.A1) and quantified.

### Gelatin & collagen zymography

Cells were treated with either DMSO or daraxonrasib in the media containing 0.1% FBS for 48 h. The media was collected and quantified using the Bradford assay. Electrophoresis was performed using a gel containing gelatin (Sigma, G2500) or collagen I (Gibco, A1048301). After electrophoresis, the gel was washed with 2.5% Triton X-100 three times and developed with developing solution [50mM Tris-HCl (pH 7.6) and 5mM CaCl_2_] for two days. The developed gel was stained with a staining solution (Coomassie Blue) and a destaining solution. Images were quantified using ImageJ software.

### RNA extraction and qPCR

RNA was extracted using an RNeasy RNA Purification Kit (Qiagen, Hilden, Germany). The extracted RNA was quantified using a NanoDrop. The isolated mRNA was converted to cDNA using a LunaScript cDNA synthesis kit from NEB. Real-time PCR was performed using the Power SYBR Green PCR master mix (Thermo Fisher Scientific). Primer sequences used for qPCR are described in the primer table below. Ct values were obtained by performing qPCR using QuantStudio 7 Flex (Applied Biology Systems). The 2^ΔΔCt^ method was used to examine the relative mRNA expression.

### Primer table

**Table:** 

Target gene	Forward primer	Reverse primer
GAPDH	TGCACCACCAACTGCTTAGC	GGCATGGACTGTGGTCATGAG
MMP1	ATGCGAACAAATCCCTTCTACC	TTTCCTCAGAAAGAGCAGCATCG
MMP2	TTGACGGTAAGGACGGACTC	ACTTGCAGTACTCCCCATCG
MMP9	GAGACCGGT GAG CTG GAT	TACACGCGAGTGAAGGTG AG
CCN2	CTTGCGAAGCTGACCTGGAAGA	CCGTCGGTACATACTCCACAGA
CCN1	GGAAAAGGCAGCTCACTGAAGC	GGAGATACCAGTTCCACAGGTC
CCND1	CCTGTCCTACTACCGCCTCA	CAGTCCGGGTCACACTTGA
RASA1	GGCCGGGAAGAAGATCCAC	GCAGACTTGACCAACTGTCATT

### Immunoblots

HOS and HOS-143 cells were seeded in 60-mm dishes (4.0 × 10^5^). The following day, cells were incubated with DMSO or daraxonrasib (5 and 10 nM) for 24 h. After treatment, cells were harvested and lysed using RIPA lysis buffer containing protease and phosphatase inhibitors. Cell lysates were normalized using the BCA protein assay kit (Thermo Fisher Scientific). After quantification of samples, the samples were mixed with 6x loading sample buffer containing 5% β-mercaptoethanol and boiled at 95°C for 10 min. SDS-PAGE was then performed and the proteins were transferred onto a PVDF membrane (Thermo Fisher, USA). After transfer, the membrane was incubated with blocking buffer (5% skim milk in TBST) for 1 h. After blocking, the membranes were incubated with target antibodies overnight at 4°C in shaking incubator. The following day, the membrane was washed by TBST three times and incubated with secondary antibody in TBST containing 1% skim milk. After secondary antibody incubation, an ECL solution (Thermo Fisher) was applied to the membrane and the target bands were detected using LI-COR. The following antibodies were used: KRAS (Proteintech, 12063–1-AP), β-actin (Santa Cruz Technology, SC-47778), p-ERK1/2 (Cell Signaling Technology, 9101S), ERK1/2 (Cell Signaling Technology, 9102), GAPDH (Cell Signaling Technology, 5174S), p-AKT S473 (Cell Signaling Technology, 4060S), AKT (Cell Signaling Technology, 4691S), ETS1 (Cell Signaling Biotechnology, 14069S), α-tubulin (Cell Signaling Technology, 3873S), and RASA1 (Proteintech, 12935–1-AP).

### Transfection

HOS cells were seeded at 8,000 cells per well in a 96-well plate. The following day, HOS cells were transfected with scrambled siRNA (5’- UUCUCCGAACGUGUCACG-3’) or RASA1 siRNA (5’-GCAGGCAGGGAAGUCUGGCAGUUAU-3’) using Lipofectamine 3000 (Invitrogen, L3000001). Both si-Scramble and si-RASA1 were purchased from Horizon Biosciences.

### GTP-KRAS pull down assay

The Active Ras Pull-Down and Detection Kit (cat # 16117, ThermoFisher) was used to assess GTP-Bound KRAS levels in HOS cells. The cells were seeded at a density of 6 × 10⁵ cells per 10-cm dish. The following day, HOS cells were transfected with scrambled siRNA or RASA1 and incubated for 48 hours. Following a wash with cold TBS, all steps were carried out in accordance with the manufacturer’s protocol (Active Ras Pull-Down and Detection Kit). HOS-143B cells were also included as a positive control for GTP-bound KRAS detection.

### Statistical analysis

All data are presented as mean ± standard deviation from three independent samples. Student’s t-test was conducted to assess the significance of two-group comparisons. For comparisons involving more than three groups, one-way ANOVA was performed, followed by Dunnett’s test for post-hoc analysis.

## Supporting information

S1 Fig Structural characteristics of daraxonrasib and basal KRAS expression in HOS and HOS-143B cells. (A) Structure of daraxonrasib, which contains a CYPA-binding domain. (B) Basal KRAS levels in HOS and HOS-143B cell lines. All experiments were conducted independently in triplicate, and the data are presented as the mean ± SD. Student’s T-tests were used to determine statistical significance. *p < .05, **p < .01, ***p < .001.
(TIF)

S1 Data Raw data. (PDF)
