# Supplementary figures and images for: Daraxonrasib, a pan-RAS inhibitor, selectively inhibits osteosarcomas with activated KRAS by halting AKT signaling and matrix metalloprotease activity
Source: PLoS One. 2025 Aug 8;20(8):e0329946. doi: 10.1371/journal.pone.0329946 (PMC12333986; doi:10.1371/journal.pone.0329946)

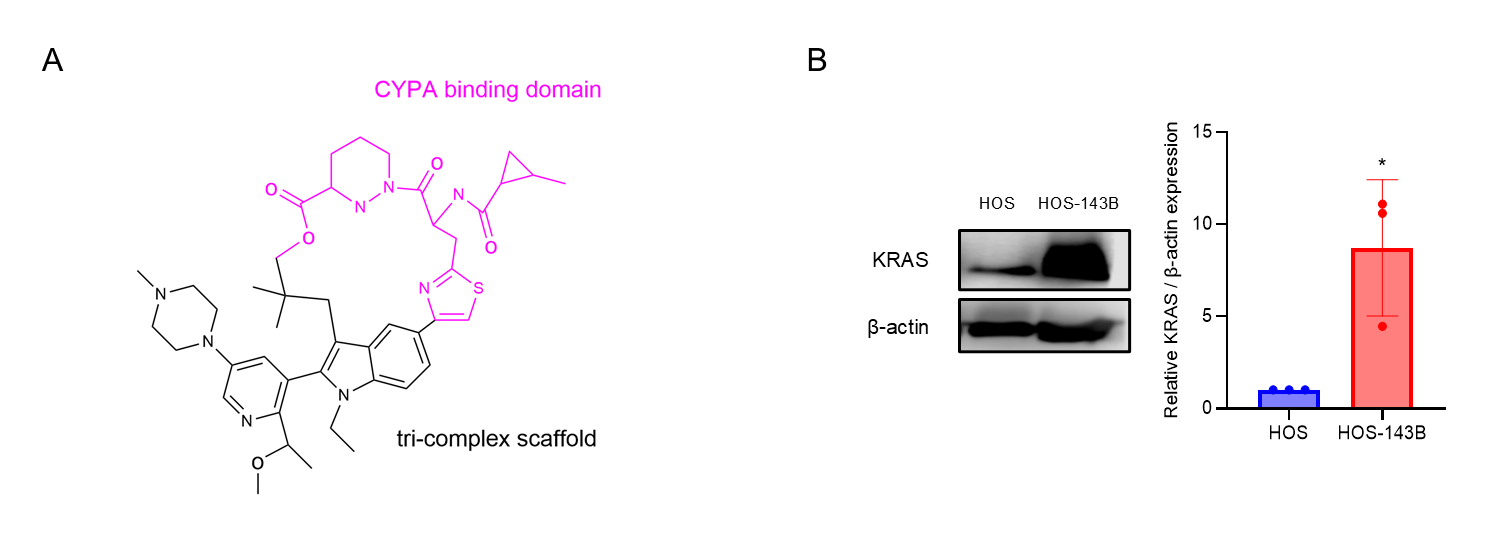

Supplement: S1 Fig — (A) Structure of daraxonrasib, which contains a CYPA-binding domain. (B) Basal KRAS levels in HOS and HOS-143B cell lines. All experiments were conducted independently in triplicate, and the data are presented as the mean ± SD. Student’s T-tests were used to determine statistical significance. *p < .05, **p < .01, ***p < .001. (TIF) [file pone.0329946.s001.tif]
