# Supplementary material for: Daraxonrasib, a pan-RAS inhibitor, selectively inhibits osteosarcomas with activated KRAS by halting AKT signaling and matrix metalloprotease activity
Source: PLoS One. 2025 Aug 8;20(8):e0329946. doi: 10.1371/journal.pone.0329946 (PMC12333986; doi:10.1371/journal.pone.0329946)

Raw data

Figure 1, Colony formation assay

HOS-143B

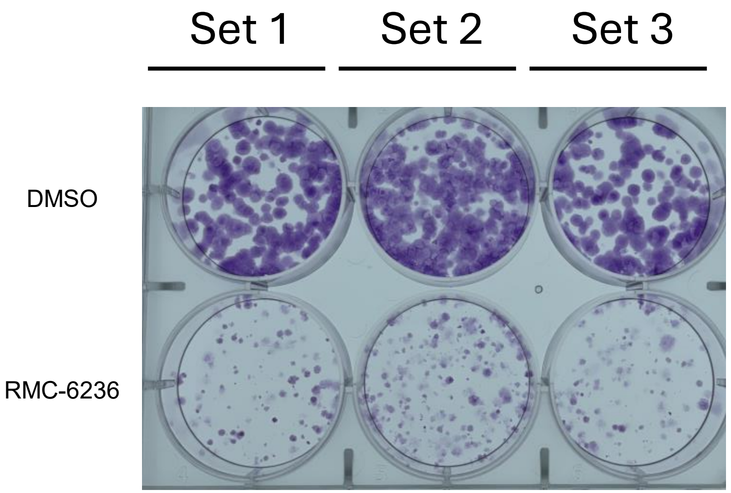

HOS

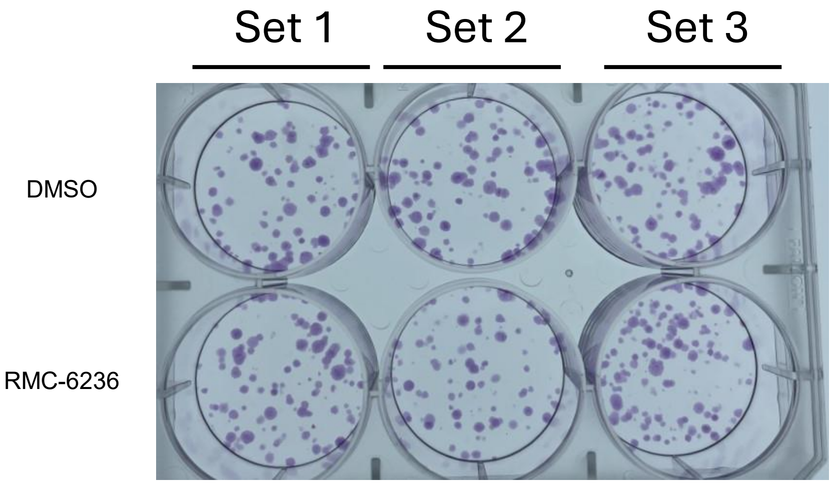

Figure 3, gelatin zymography

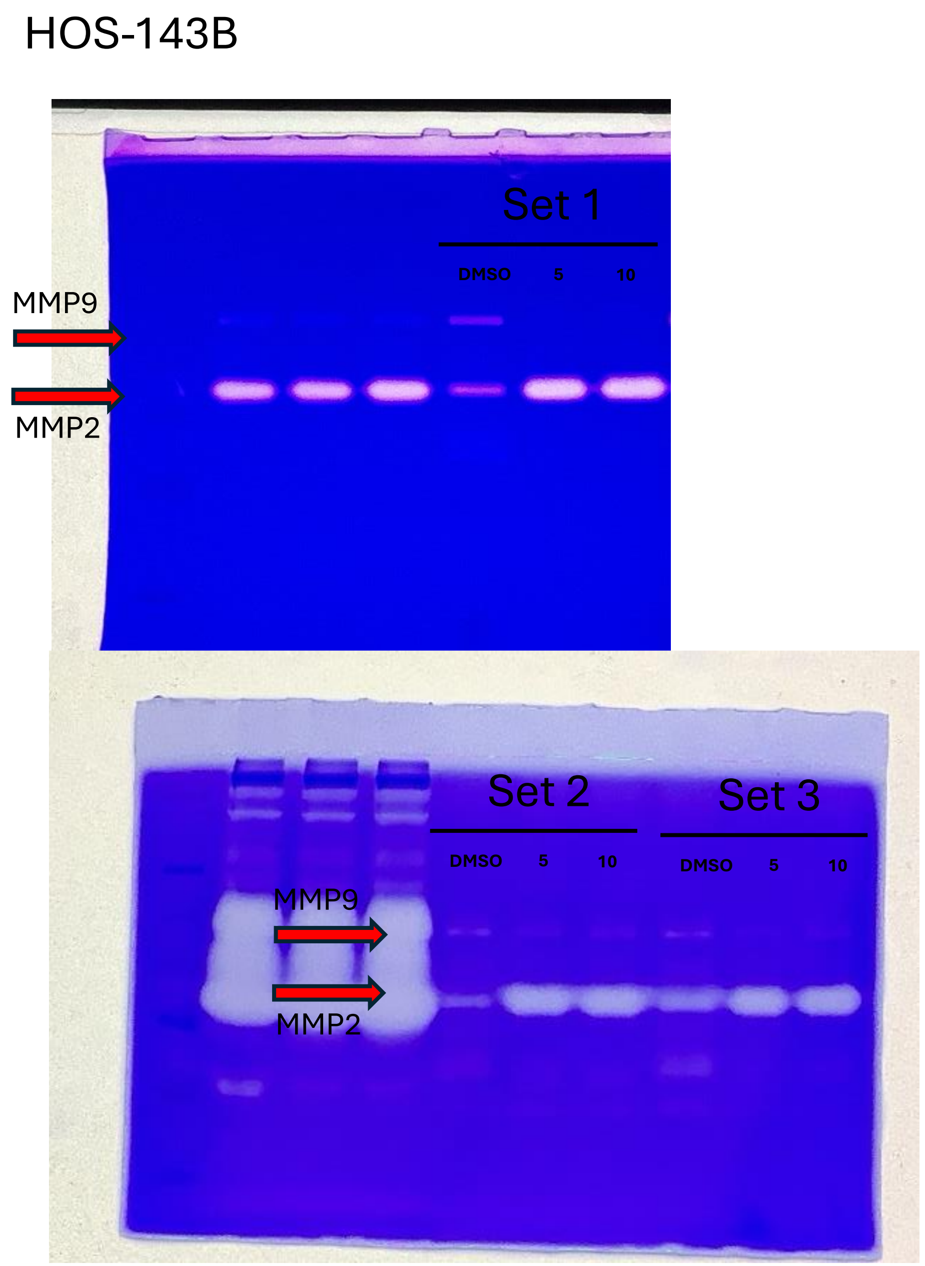

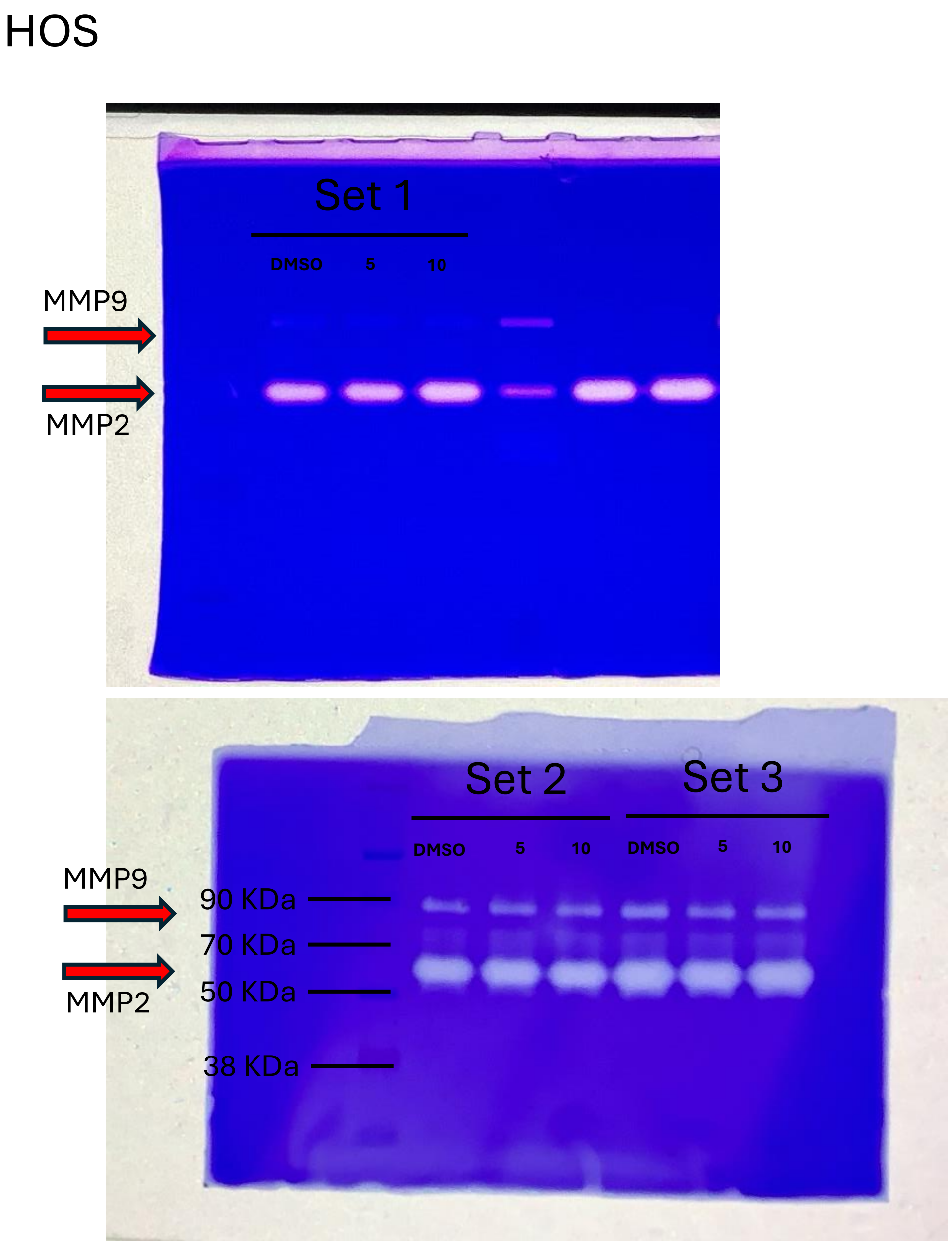

Figure 4, collagen zymography

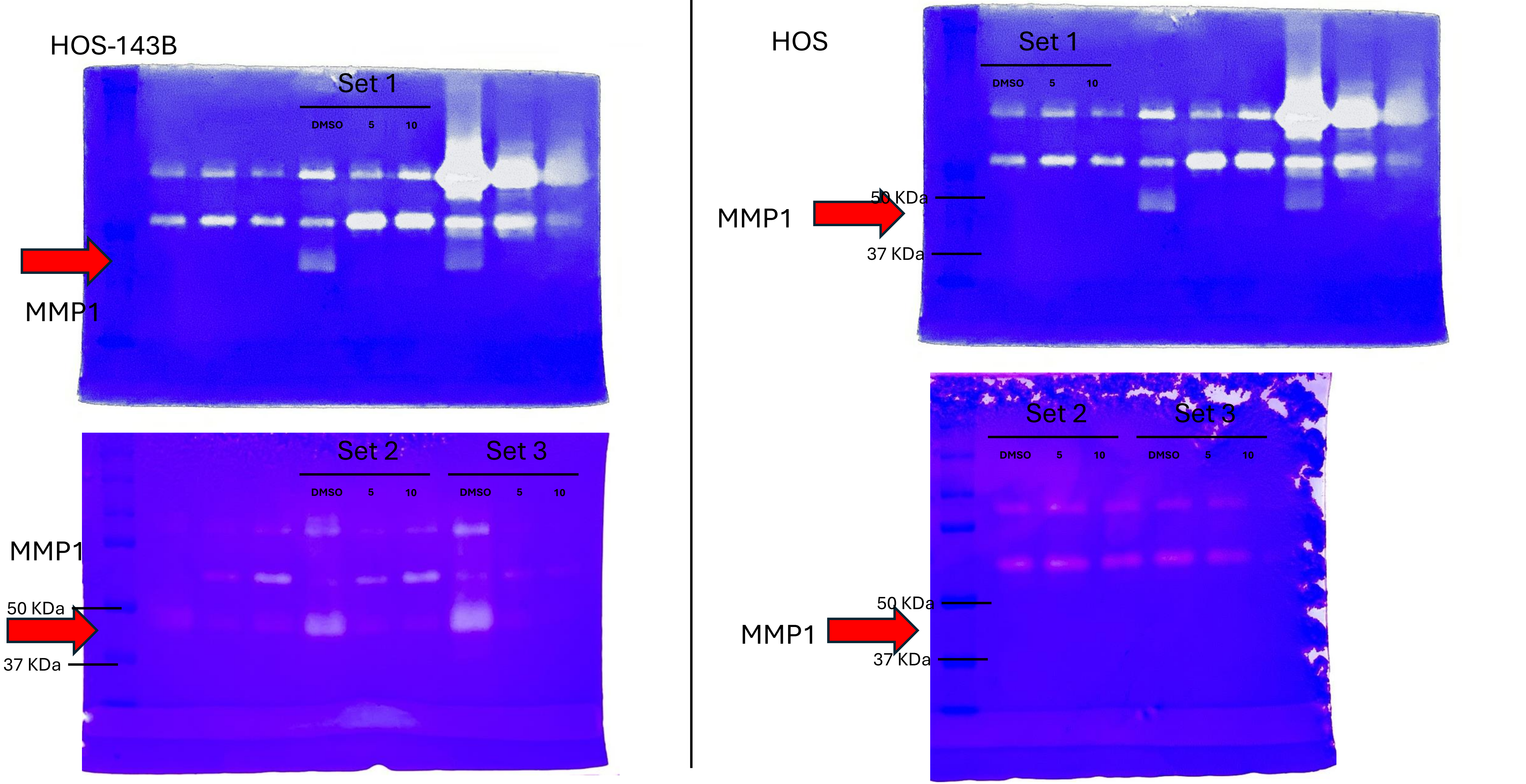

Figure 5, Western blot

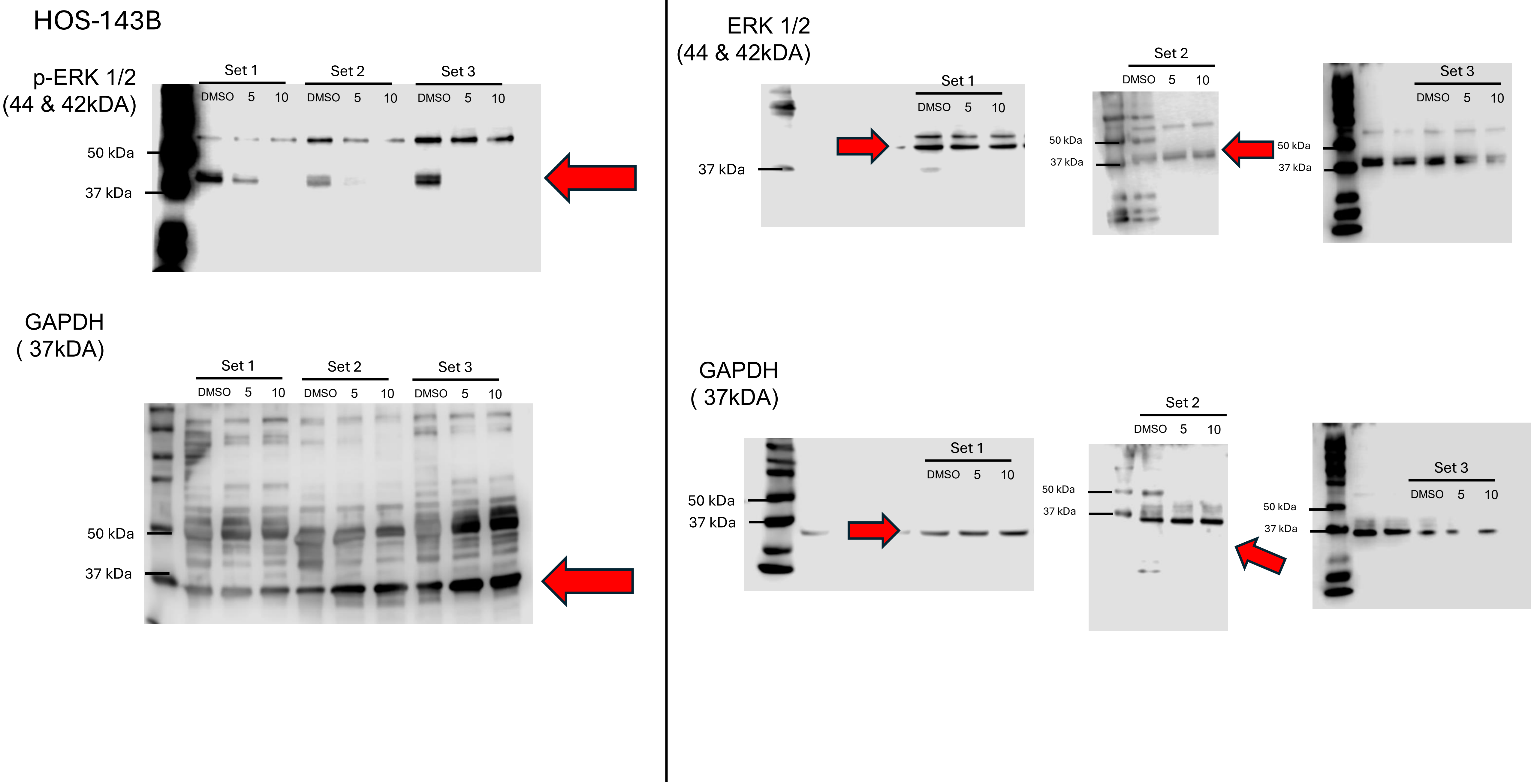

Figure 5, Western blot

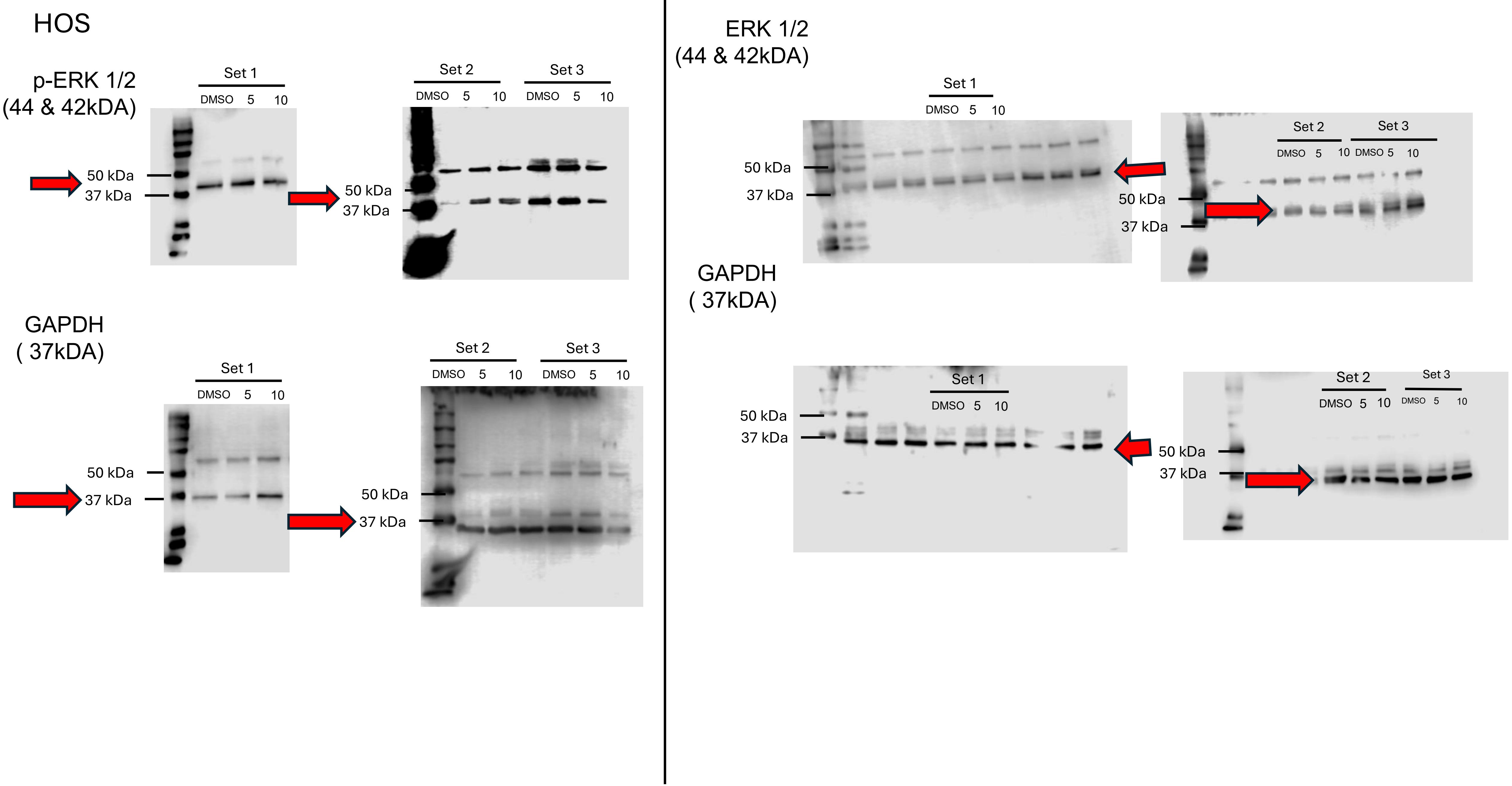

Figure 5, Western blot

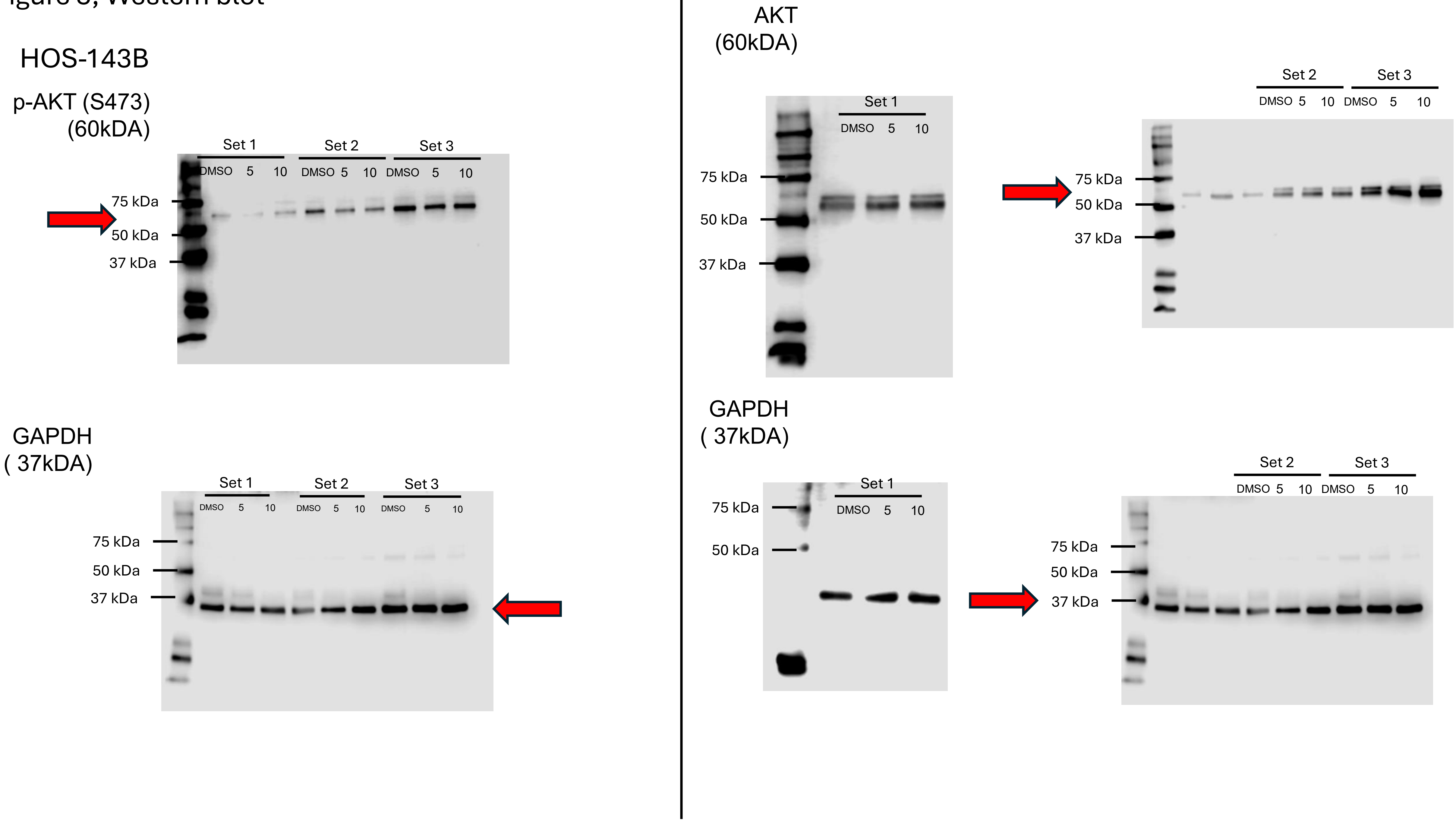

Figure 5, Western blot

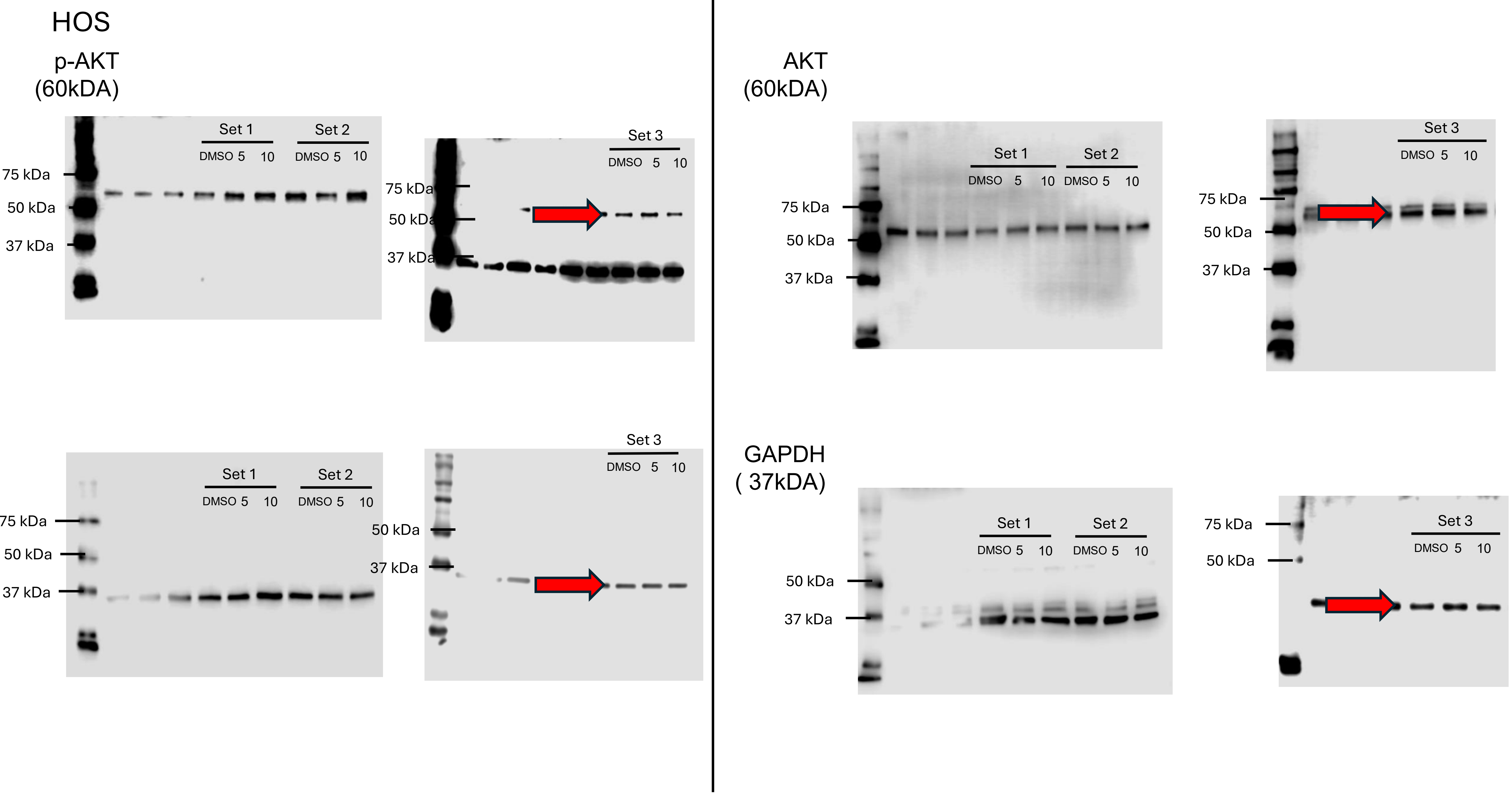

Figure 5, Western blot

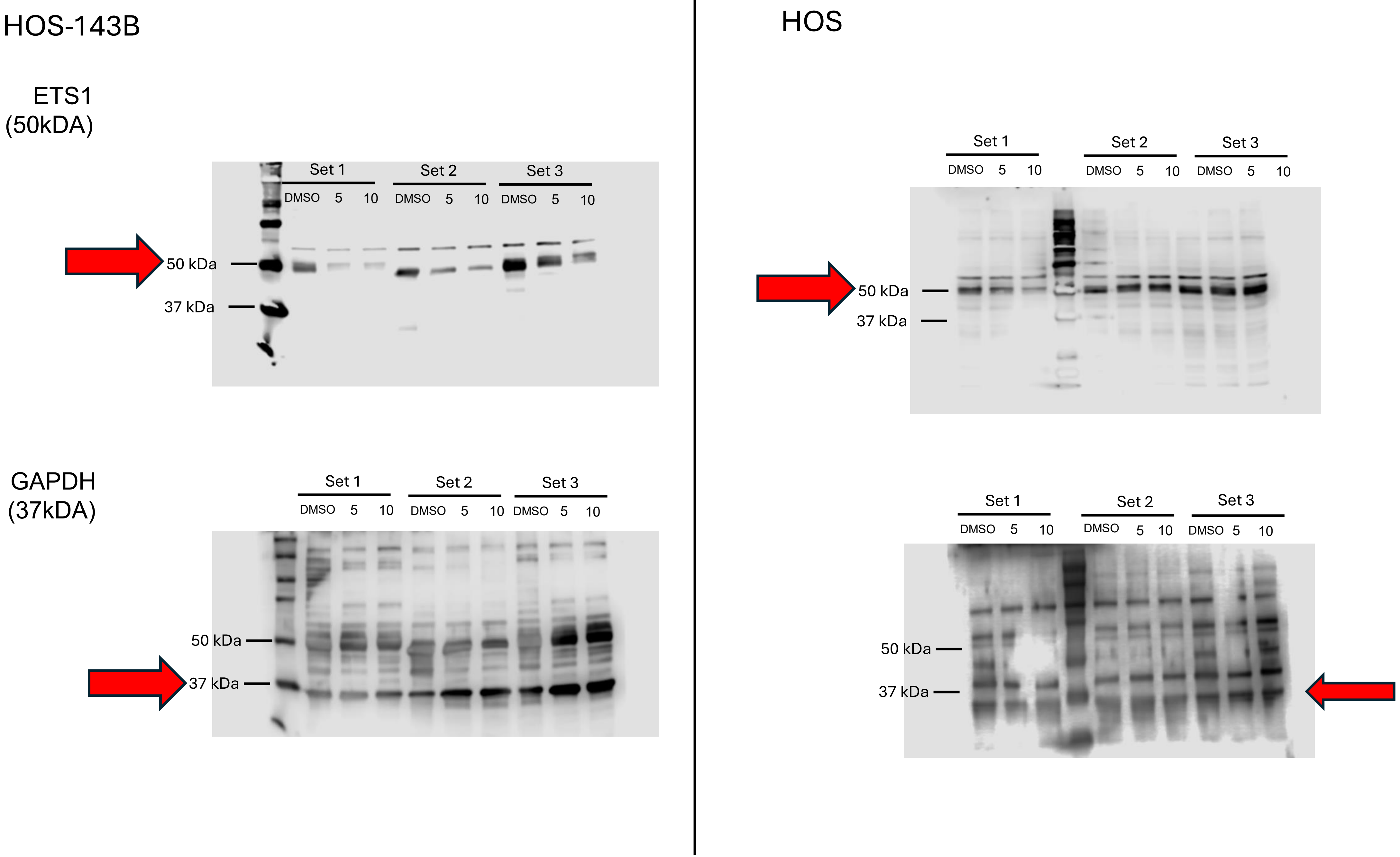

Figure 6, si scramble and si RASA1 WB in HOS cell line

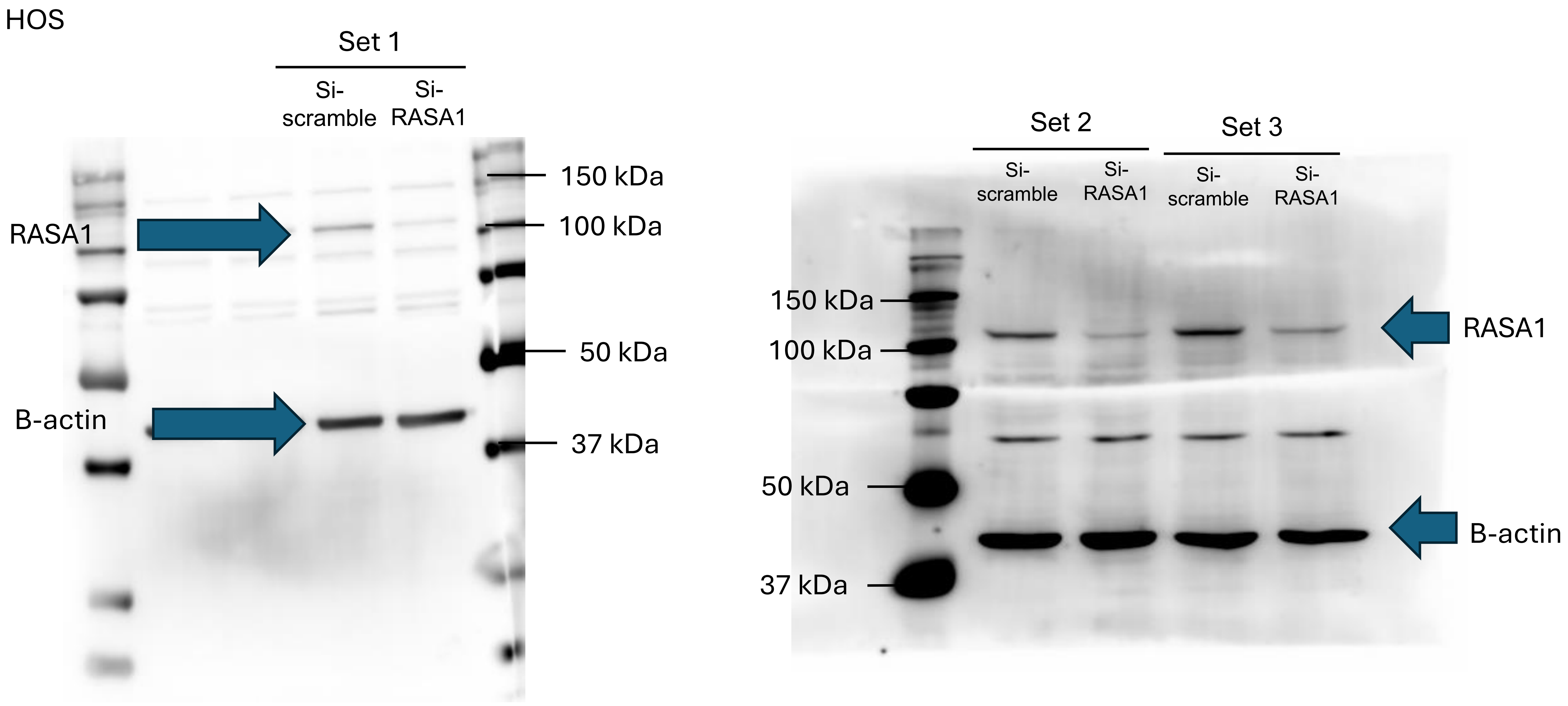

Figure 6, GTP-KRAS pull down assay 1st set

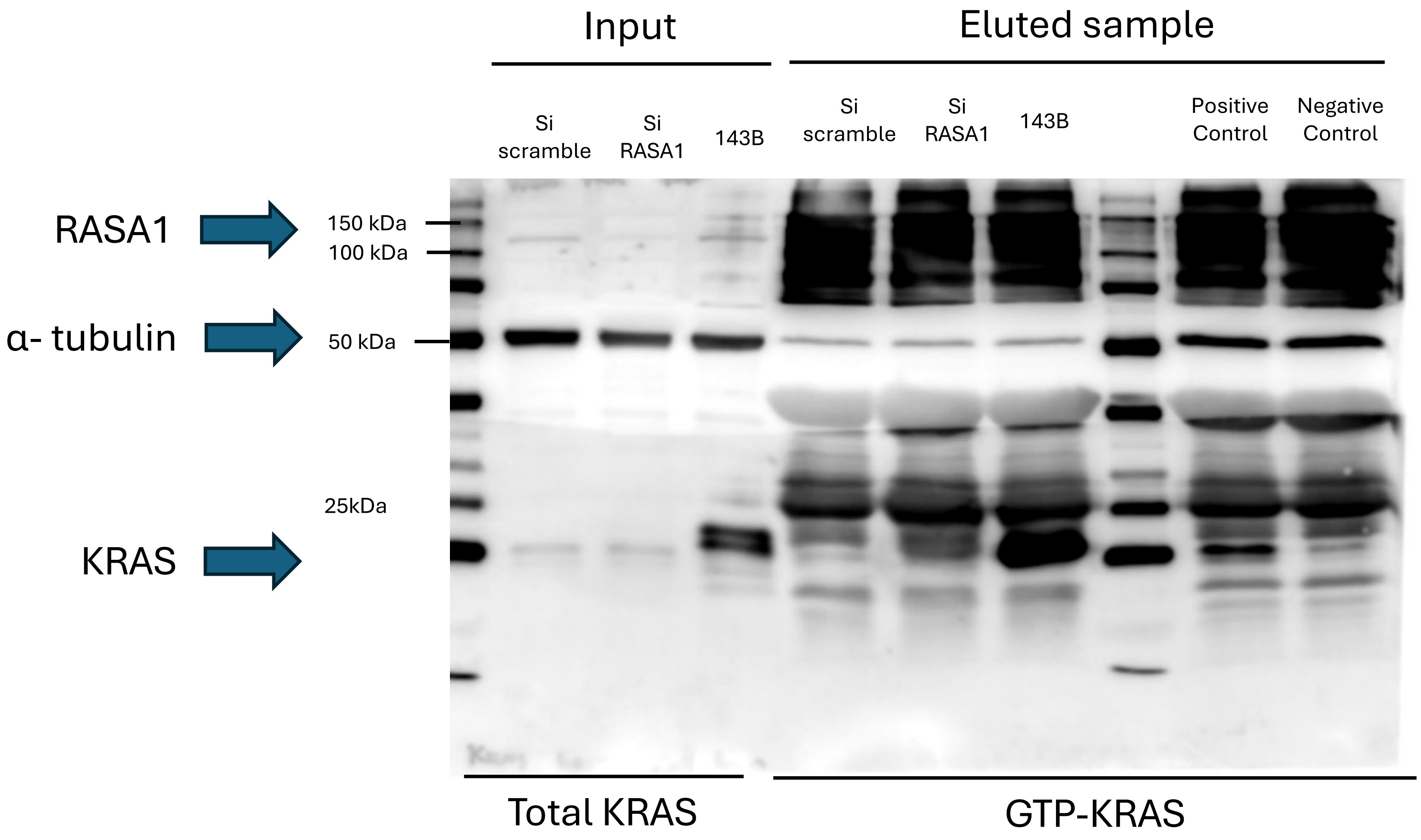

Figure 6, GTP-KRAS pull down assay 2nd set

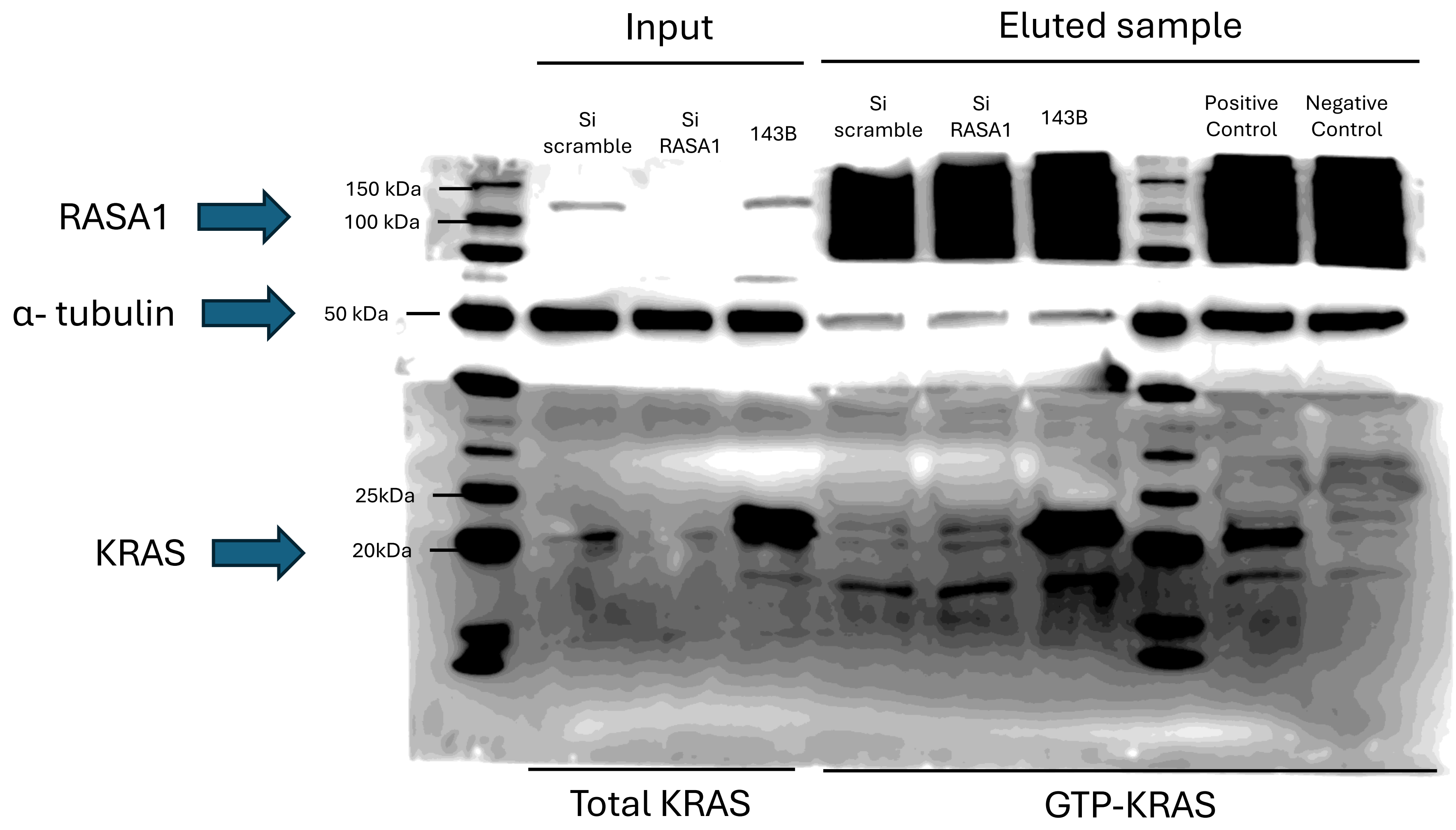

Figure 6, GTP-KRAS pull down assay 3rd set

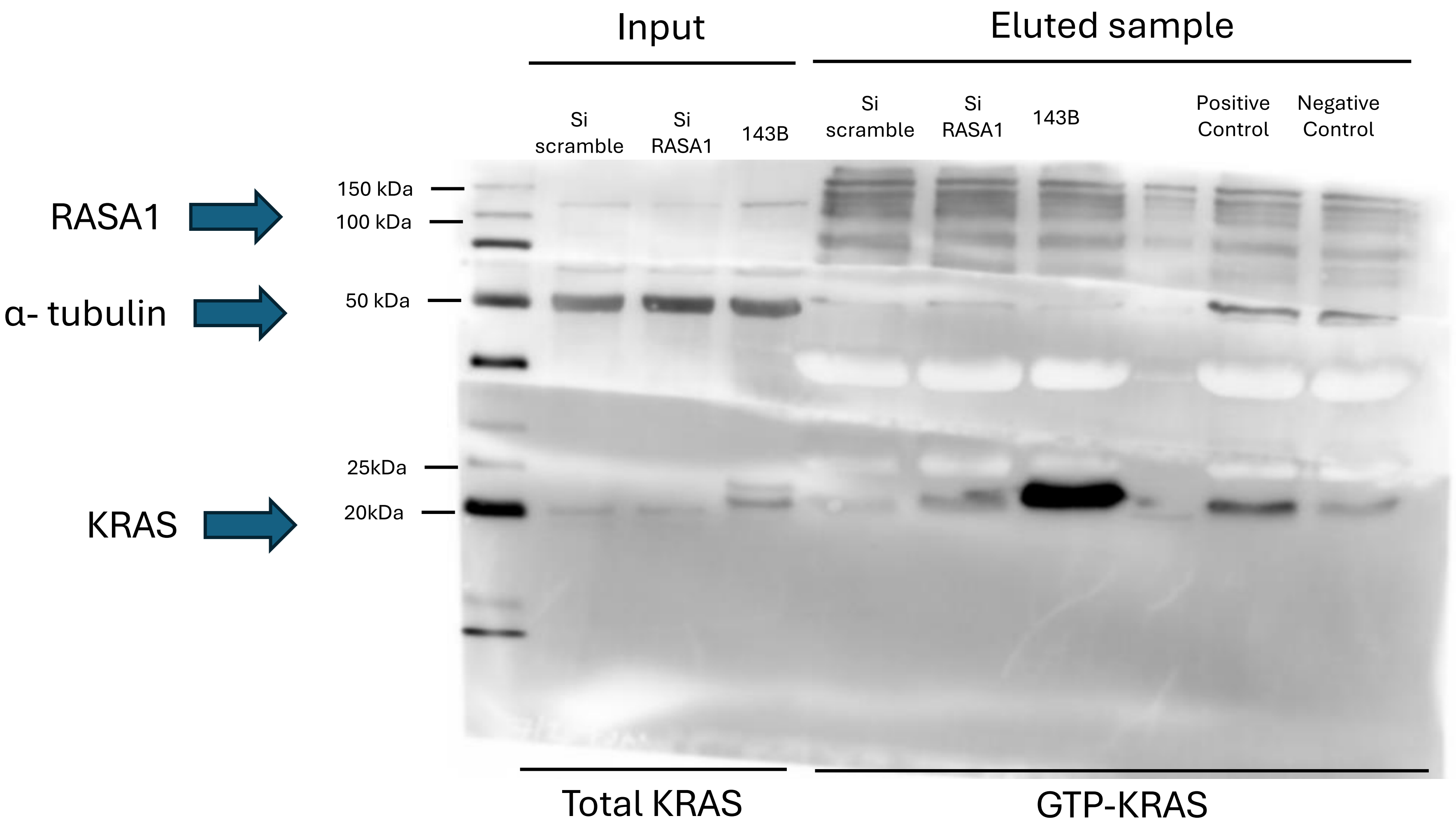

Supplementary Figure 1, Western blot

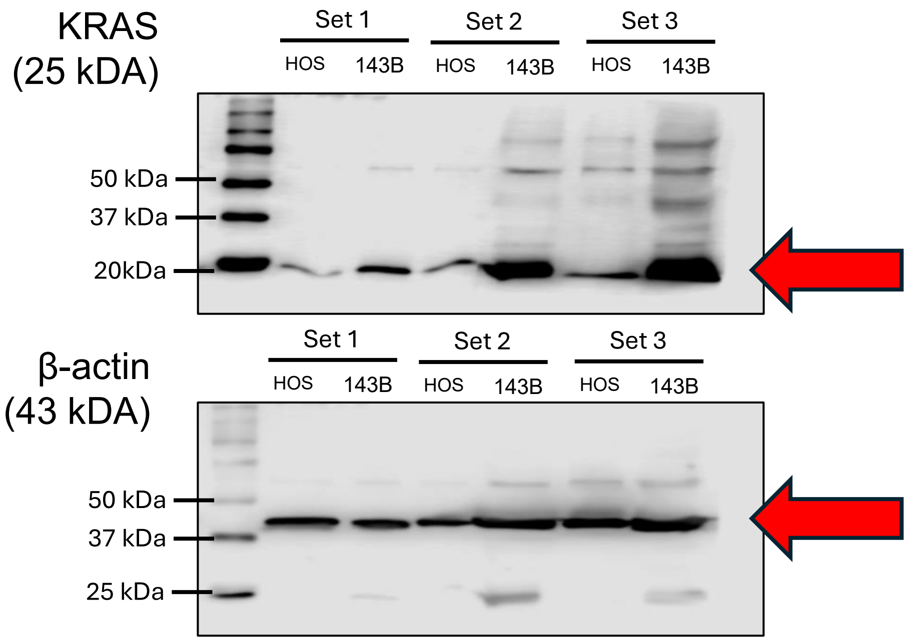

Supplement: S1 Data — (PDF) [file pone.0329946.s002.pdf]
